# Creep Crack Growth Behavior during Hot Water Immersion of an Epoxy Adhesive Using a Spring-Loaded Double Cantilever Beam Test Method

**DOI:** 10.3390/ma16020607

**Published:** 2023-01-08

**Authors:** Kota Nakamura, Yu Sekiguchi, Kazumasa Shimamoto, Keiji Houjou, Haruhisa Akiyama, Chiaki Sato

**Affiliations:** 1 Department of Mechanical Engineering, Tokyo Institute of Technology, 4259 Nagatsuta-cho, Midori-ku, Yokohama 226-8503, Japan; 2 Institute of Innovative Research, Tokyo Institute of Technology, 4259 Nagatsuta-cho, Midori-ku, Yokohama 226-8503, Japan; 3 Nanomaterials Research Institute, National Institute of Advanced Industrial Science and Technology (AIST), 1-1-1 Higashi, Tsukuba 305-8565, Japan

**Keywords:** adhesive bonding, hydrothermal creep, degradation, creep fracture toughness, aging

## Abstract

Double cantilever beam (DCB) tests were conducted by immersing the specimens in temperature-controlled water while applying a creep load using a spring. By introducing a data reduction scheme to the spring-loaded DCB test method, it was confirmed that only a single parameter measurement was sufficient to calculate the energy release rate (ERR). Aluminum alloy substrates bonded with an epoxy adhesive were used, and DCB tests were performed by changing the initial load values, spring constants, and immersion temperatures for two types of surface treatment. The initial applied load and spring constant had no effect on the ERR threshold. In contrast, the threshold decreased with the increasing immersion temperature, but even in the worst case, it was 15% of the critical ERR in the static tests. Using the creep crack growth relationship, it was revealed that there were three phases of creep immersion crack growth in the adhesive joints, and each phase was affected by the temperature. The spring-loaded DCB test method has great potential for investigating the combined effects of creep, moisture, and temperature, and this study has demonstrated the validity of the test method. The long-term durability of adhesive joints becomes increasingly important, and this test method is expected to become widespread.

## 1. Introduction

Joining technology is essential for the assembly of parts. In particular, adhesive bonding is superior to other joining methods in terms of stress dispersion, bonding of dissimilar materials, and weight reduction. Therefore, they are widely used for electrical parts, vehicles, and buildings. As the use of adhesives increases, reliability issues become more important. Many factors are related to the degradation of adhesive joints, but moisture is one of the most well-known and important factors [1,2]. Because adhesives are mainly composed of polymers, they absorb water. Water penetrating the adhesive not only changes the material properties of the adhesive [3,4] but also attacks the interface and weakens the bond [5,6,7]. Creep and fatigue loads are also important for the durability of adhesive bonding [8,9,10]. The creep strength and fatigue strength are much lower than the static strength but even lower under humid conditions. Therefore, it is important to study the combination of moisture and creep or fatigue loads [11]. In general, specimens are first immersed or subjected to high humidity, and then fatigue tests are conducted under ambient conditions [12,13,14,15]. Conversely, some studies have found that the deterioration of joints by immersion in water is accelerated by external loads [3,16,17,18,19]. Therefore, immersion in water during creep/fatigue testing could alter the results.

The mechanical properties of adhesive joints are generally evaluated based on their strength and toughness. The best-known method for evaluating strength is the single-lap joint (SLJ) test. Springs are sometimes used when applying creep loads to SLJ specimens under humid conditions [20,21]. Spring-loaded SLJ specimens have also been immersed in hot water or exposed on ship decks [16,22]. The double cantilever beam (DCB) test is commonly used to evaluate fracture toughness [23,24]. In the static fracture test, the DCB specimen is set to a mechanical tensile testing machine and loaded in mode I at a constant opening speed. Three parameters, the crack length, load, and opening displacement, are required to calculate the fracture toughness of the adhesives in the DCB tests. However, the optical measurement of the crack length causes large errors in the calculation of fracture toughness. Therefore, new approaches for crack measurement methods using digital image correlation and mechanoluminescence have been proposed [25,26]. Moreover, a method to reduce one of the three parameters has been proposed: the compliance-based beam method (CBBM) [27]. To apply a creep load to the DCB specimens, a tensile testing machine can be used in the same way as for static tests [8]. However, in this case, it is difficult to maintain a specimen exposed to humid conditions. Another method of testing creep crack resistance is to insert a wedge into the DCB specimen, which is the Boeing wedge test [28,29,30,31]. In this case, creep-loaded specimens can be exposed to different environmental conditions to investigate their degradation [32,33,34,35]. Conversely, optical crack measurement is required to calculate the fracture toughness, leading to concerns regarding increased calculation errors. Studies have been conducted to accurately measure the crack length in wedge tests [36,37]; however, these techniques are not a panacea. To overcome these weaknesses, a spring-loaded DCB test has been proposed for hot water immersion tests but remains largely untested to date [38]. Similar to the spring-loaded SLJ test, the spring exerts a creep load on the specimen; however, the displacement increases and the load decreases as the crack propagates. By measuring the load and displacement over time and applying the CBBM, a change in the fracture toughness can be obtained. Therefore, it has the potential to evaluate crack resistance under a combination of creep load and immersion without measuring the crack length. Although no studies have focused on the effectiveness and accuracy of the spring-loaded DCB test method so far, this test method deserves more attention.

In this study, spring-loaded DCB tests were conducted under hot and wet conditions. First, the effects of initial load and spring constant on the creep crack growth (CCG) were investigated to verify the accuracy of the calculation process of the energy release rate and the experimental equipment in the test method. In addition, the effects of the water temperature and surface preparation on CCG behavior were investigated. Three temperature conditions (32, 63, and 90 °C) and two surface treatments (sandblasting and pickling) were examined, and the results are discussed in terms of the CCG rate.

## 2. Methods

### 2.1. Compliance-Based Fracture Energy Calculation

The fracture behavior of adhesive joints has been widely discussed based on linear elastic fracture mechanics, and the mode I critical energy release rate, $G_{\mathrm{IC}}$, is given by:

(1) $$G_{\mathrm{IC}}=\frac{P^{2}}{2b}\frac{dC}{da}$$

where P is the applied load, b is the specimen width, C=δ/P is the compliance, δ is the opening displacement, and *a* is the crack length. Therefore, in DCB tests, the crack length, displacement, and load must be measured to calculate the energy release rate. However, because it is known that simple beam theory (SBT) provides the relationship between these three parameters, a parameter reduction scheme using SBT (i.e., CBBM) was validated for measuring the fracture toughness of various adhesives [39,40,41]. Considering the shear effect in the SBT, the compliance can be expressed as follows:

(2) $$C=\frac{\delta }{P}=\frac{8a_{e}^{3}}{Ebh^{3}}+\frac{12a_{e}}{5bhG}$$

where $a_{e}$ is the equivalent crack length, which is the sum of the crack length a and the crack length correction Δa; E, G, and h are the longitudinal and transverse moduli of the elasticity and thickness of the substrate, respectively. Substituting Equation (2) into Equation (1):

(3) $$G_{\mathrm{IC}}=\frac{6P^{2}}{b^{2}h}\left(\frac{2a_{e}^{2}}{h^{2}E}+\frac{1}{5G}\right)$$

is obtained. By solving Equation (2) for $a_{e}$ and substituting it into Equation (3), the energy release rate is expressed as a function of the load and displacement [40].

### 2.2. Load–Displacement Curve in the Spring-Loaded Testing Method

**Figure 1:**
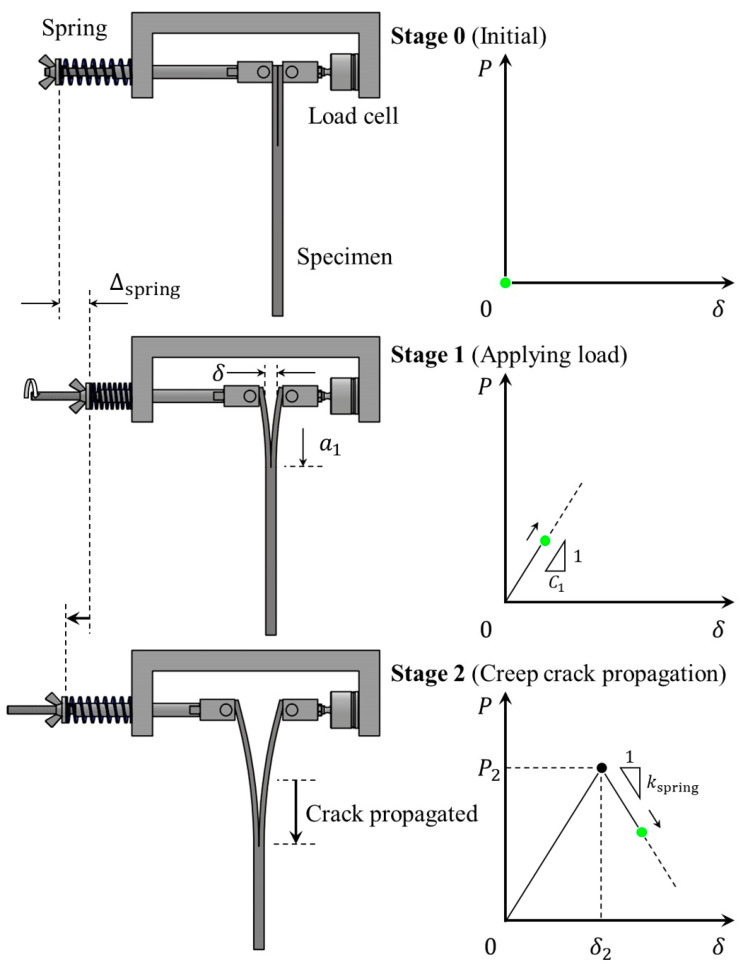
Schematic diagram of a spring-loaded DCB test: stage 0—before a test; stage 1—load applying process by tightening a nut; stage 2—creep load applying process.

A schematic of the loading procedures in the spring-loaded DCB test method is shown in Figure 1. The wing nut was tightened to compress the spring and apply an initial load to the specimen. Here, the amount of spring contraction, $\Delta_{\mathrm{spring}}$, is given by:

(4) $$\Delta_{\mathrm{spring}}=\frac{P}{k_{\mathrm{spring}}}$$

where $k_{\mathrm{spring}}$ is the spring constant. The spring-loaded DCB test includes two stages: load applying stage (stage 1) and crack propagation stage (stage 2). In the first stage, the crack length is considered constant as $a=a_{1}$, where $a_{1}$ is the crack length at the beginning of the first stage. Therefore, the compliance in the first stage $C_{1}$ keeps constant. From Equations (2) and (4), the opening displacement in the first stage can be expressed as

(5) $$\delta =PC_{1}=k_{\mathrm{spring}}\Delta_{\mathrm{spring}}C_{1}$$

Thus, there is a linear relationship between *δ* and $\Delta_{\mathrm{spring}}$. In the second stage, the cracks gradually propagate. Therefore, the compliance and displacement increases and the load decreases. Considering the change in the magnitude of the spring contraction, the relationship between the load and displacement is as follows:

(6) $$\left(P-P_{2}\right)=-k_{\mathrm{spring}}\left(\delta -\delta_{2}\right)$$

where $P_{2}$ and $\delta_{2}$ are the load and displacement at the stage change point, i.e., the initial values of the second stage.

## 3. Experimental

### 3.1. Materials and Specimens

**Figure 2:**
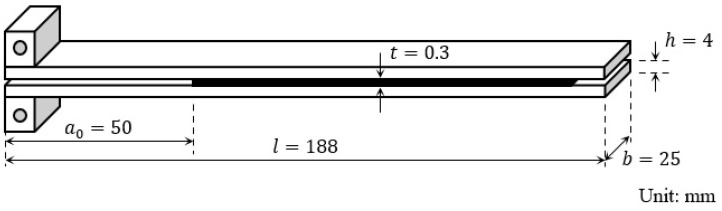
Geometry of a DCB test specimen.

An aluminum alloy (A6061-T6) with a length of l=188 mm, width of b=25 mm, and thickness of h=4 mm was used as the substrate of the DCB specimens. The thermoset epoxy adhesive used (Cemedine Co., Ltd., Tokyo, Japan) consisted of bisphenol A epoxy resin, which contained carboxyl-terminated butadiene acrylonitrile rubber (CTBN), fumed silica, CaCO_3_, and CaO as a base resin; dicyandiamide as a curing agent; and 3-(3,4-dichlorophenyl)-1,1’-dimethylurea as a curing accelerator. CTBN was contained to improve its fracture toughness and elongation. Two different surface treatments were applied to the aluminum alloy substrates: sandblasting and pickling. The process of the first method was sandblasting with an air pressure of 0.7 MPa using Al_2_O_3_ abrasive grains, followed by degreasing with acetone. In the second method, the substrates were degreased with acetone, immersed in an alkaline solution at 60 °C for 30 s, washed with purified water, dried, and immersed in an acidic solution at 60 °C for 30 s. The substrates were bonded with the adhesive and cured for 1 h at 180 °C in an electric furnace. The thickness of the adhesive layer was controlled by inserting a 0.3 mm thick polytetrafluoroethylene (PTFE) sheet at both ends of the adhesive layer. The PTFE sheet also produced an initial crack: $a_{0}=50$ mm. The geometry of the DCB specimens used in this study is shown in Figure 2. In addition, before the creep test, a crack of approximately 5 mm in length was generated using a universal tensile tester (AGS-X 10kN, Shimadzu Corp., Kyoto, Japan) to produce a sharp initial crack, i.e., $a_{1}\approx 55$ mm.

### 3.2. Experimental Setup

**Figure 3:**
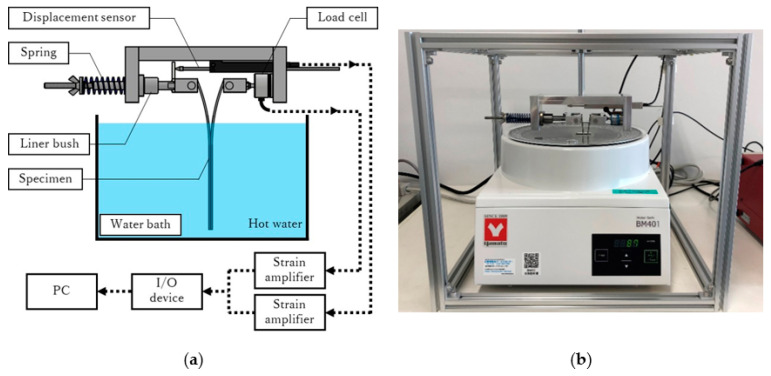
(**a**) Schematic diagram and (**b**) photograph of the spring-loaded DCB test setup.

A schematic diagram of the spring-loaded experimental setup is shown in Figure 3. The applied load and opening displacement were measured using a load cell (LUX-B-1kN, Kyowa Electronic Instruments Co., Ltd., Tokyo, Japan) and a displacement sensor (DTK-A-50, Kyowa Electronic Instruments Co., Ltd., Tokyo, Japan). These output data were converted to voltage via strain amplifiers (DA-18A, Tokyo Measuring Instruments Laboratory Co., Ltd., Tokyo, Japan), digital data via an analog input unit (AI-1608GY-USB, CONTEC Co., Ltd., Osaka, Japan), and then recorded on a PC using in-house developed data acquisition software. The temperature of the purified water was controlled using a water bath.

### 3.3. Spring-Loaded DCB Test

The tests were conducted under two test conditions with different objectives. First, the accuracy of the experimental system and calculation method was investigated by changing the spring constant and initial loading conditions. The test conditions are listed in Table 1. Next, the effects of the surface treatment and water immersion temperature, T (32, 63, and 90 °C), on the crack growth behavior were investigated keeping the initial energy release rate, $G_{\mathrm{ini}}$, at approximately 500 J/m^2^, which is approximately 75% of the critical energy release rate ($G_{\mathrm{IC}}=679\;$ J/m^2^). The test conditions are listed in Table 2.

## 4. Results and Discussion

### 4.1. Verification of the Spring-Loaded DCB Test

**Figure 4:**
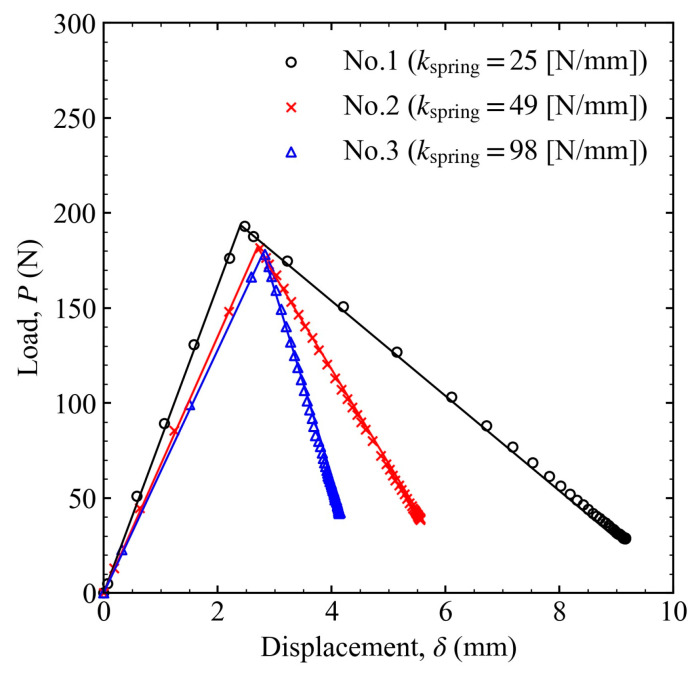
Relationship between load and displacement when the spring constant was changed to 25, 49, and 98 N/m.

**Figure 5:**
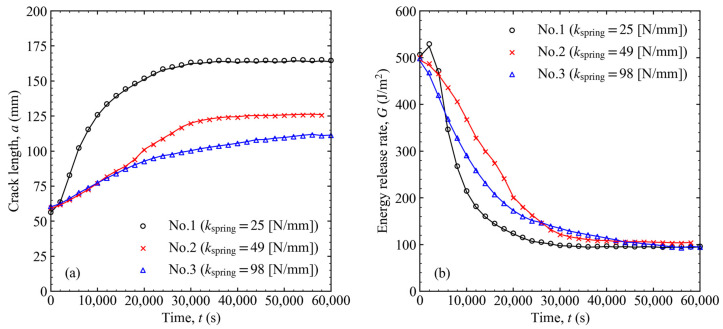
(**a**) Crack length and (**b**) energy release rate variation over time when the spring constant was changed to 25, 49, and 98 N/m.

A linear bush was inserted to reduce the friction and keep the shaft coaxial with the load cell (see Figure 3). If the friction is negligible and the shaft moves smoothly, Equations (5) and (6) are satisfied, and the load can be calculated from the displacement, and vice versa. In this way, a further parameter reduction is possible, and the energy release rate can be calculated from the load or displacement only. The results with varying spring constants are shown in Figure 4 for the load–displacement relationship and in Figure 5 for the crack length and energy release rate as a function of time. The lines indicate the results obtained with measured displacements only, whereas the marks indicate the results obtained with the measured loads and displacements. Because the difference between the lines and marks was small, it shows that the friction was sufficiently low, and the equipment system worked well. Thus, Equations (5) and (6) are valid, and it is possible to calculate the energy release rate by measuring only one parameter. However, to avoid unexpected situations, both the loads and displacements were recorded in all experiments, and the energy release rate was calculated from these two values in subsequent experiments.

**Figure 6:**
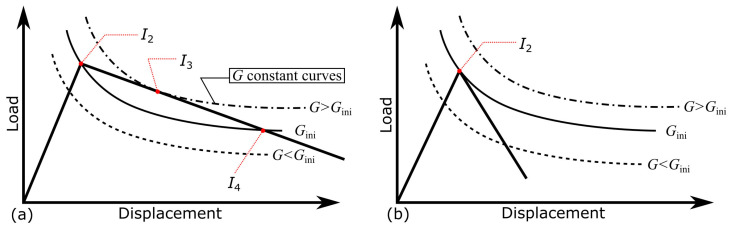
Schematic diagram of the relationship between the experimentally determined load–displacement relationship and energy release rate constant curve in the case for (**a**) a small spring constant and (**b**) a large spring constant.

When the spring constant was changed, the slopes of the load–displacement relationship in the first and second stages varied, as shown in Figure 4. The smaller the spring constant, the more cracks propagated, as shown in Figure 5a. Conversely, the energy release rate converged to a certain value regardless of the spring constant, as shown in Figure 5b. This value is called the threshold for the energy release rate ($G_{\mathrm{th}}$). This was approximately 15% of $G_{\mathrm{IC}}$. Although $G_{\mathrm{ini}}$ was set to almost the same value, different spring constants resulted in different changes in the energy release rate. At the smallest spring constant, the energy release rate initially increased and then rapidly decreased to approach $G_{\mathrm{th}}$. However, at a larger spring constant, the energy release rate decreased monotonically to $G_{\mathrm{th}}$. The difference in the initial G variation was related to the relationship between the line in Equation (6) and the curve of $G=G_{\mathrm{ini}}$. In Figure 6, $I_{2}$ is the change point from stages 1 to 2. When the spring constant was small, the line was positioned above the curve of $G=G_{\mathrm{ini}}$, between $I_{2}$ and $I_{4}$, as shown in Figure 6a. In such cases, *G* increased between $I_{2}$ and $I_{3}$ and then decreased. $I_{3}$ is the point where the line and the G constant curve intersected at a single point. Conversely, when the spring constant was sufficiently large, the line was below the curve after intersecting the curve at $I_{2}$, as shown in Figure 6b. In this case, G decreased monotonically.

**Figure 7:**
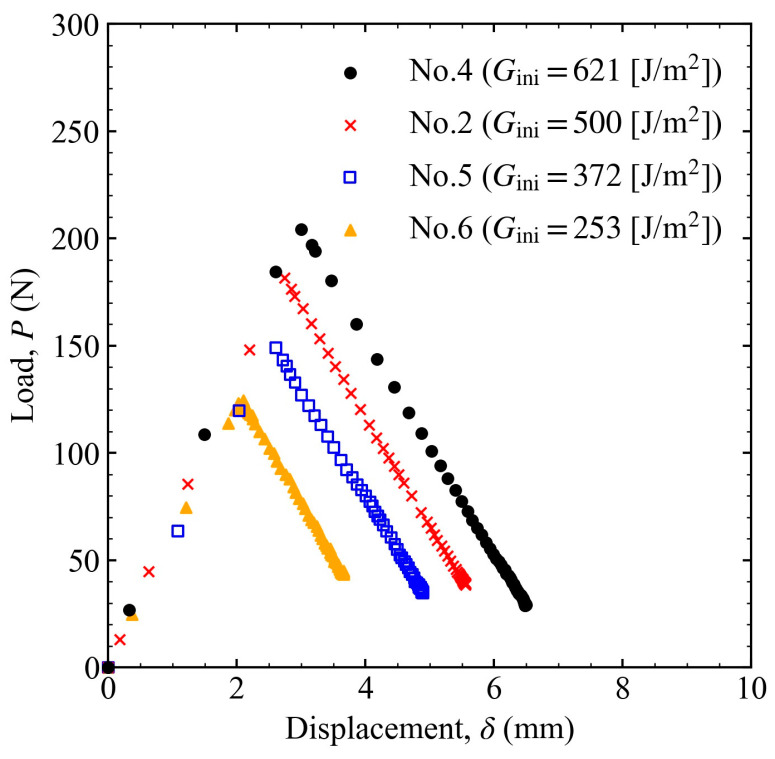
Relationship between the load and displacement when the initial load level was changed to 621, 500, 372, and 253 J/m^2^.

**Figure 8:**
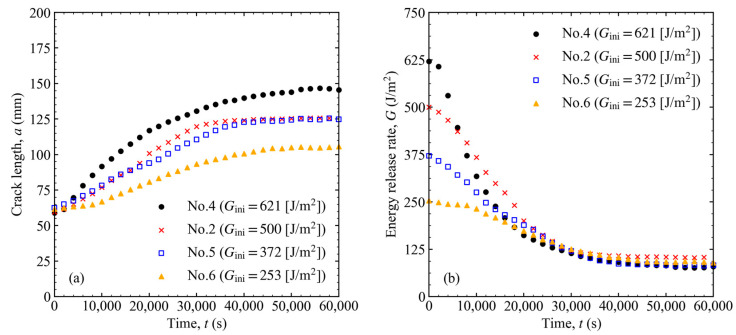
(**a**) Crack length and (**b**) energy release rate variation over time when the initial load level was changed to 621, 500, 372, and 253 J/m^2^.

The results of varying the initial energy release rate using the same spring are shown in Figure 7 for the load–displacement relationship and in Figure 8 for the crack length and energy release rate versus time. Because the spring constants were the same, the slopes of the load–displacement results were the same for different $G_{\mathrm{ini}}$. In addition, $G_{\mathrm{th}}$ was almost the same, even when $G_{\mathrm{ini}}$ varied. Therefore, the threshold was considered to be independent of the spring constant and the initial load level.

### 4.2. Effects of the Surface Treatment and Immersion Temperature

**Figure 9:**
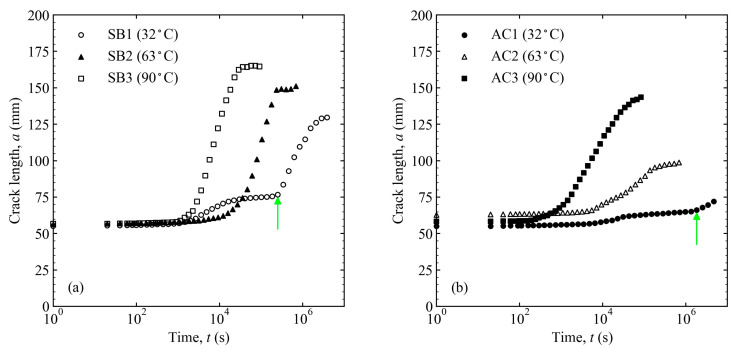
Crack length variation over time at different temperatures and surface treatments: (**a**) sandblasting and (**b**) pickling. The light-green arrows are the failure mode changing points, which relate to the locations of the light-green triangles in Figure 11.

**Figure 10:**
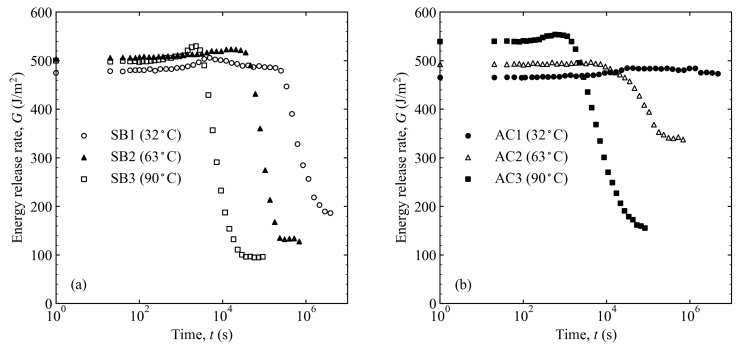
Energy release rate variation over time at different temperatures and surface treatments: (**a**) sandblasting and (**b**) pickling.

The changes in the crack length and energy release rate over time are shown in Figure 9 and Figure 10 at different temperatures and surface treatments. Comparing the crack propagation at different water temperatures, it is clear that the hotter the water, the more the crack propagated. The decrease in the energy release rate also tended to increase with the increasing water temperature. Comparing the differences in the surface preparation, it can be seen that the pickling treatment suppressed crack propagation more than the sandblasting treatment.

**Figure 11:**
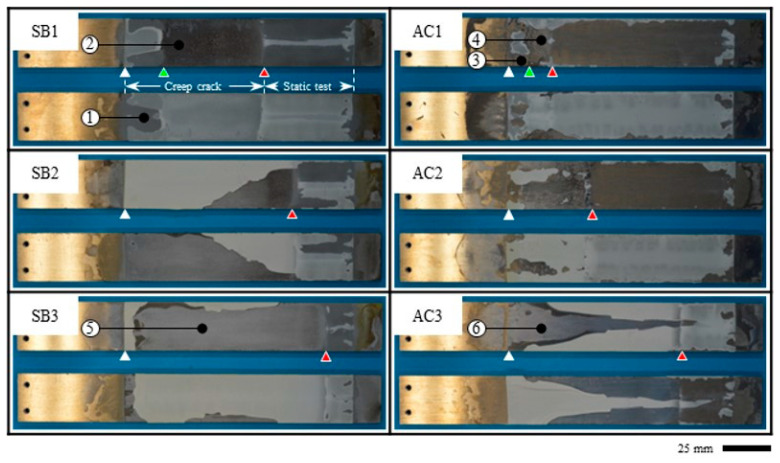
The macroscopic image of the fractured surfaces of the specimens after the spring-loaded DCB tests. White triangles: initial crack positions; light-green triangles: failure mode changing points; red triangles: crack positions after creep immersion tests. Numbered locations are magnified using a microscope in Figure 12.

**Figure 12:**
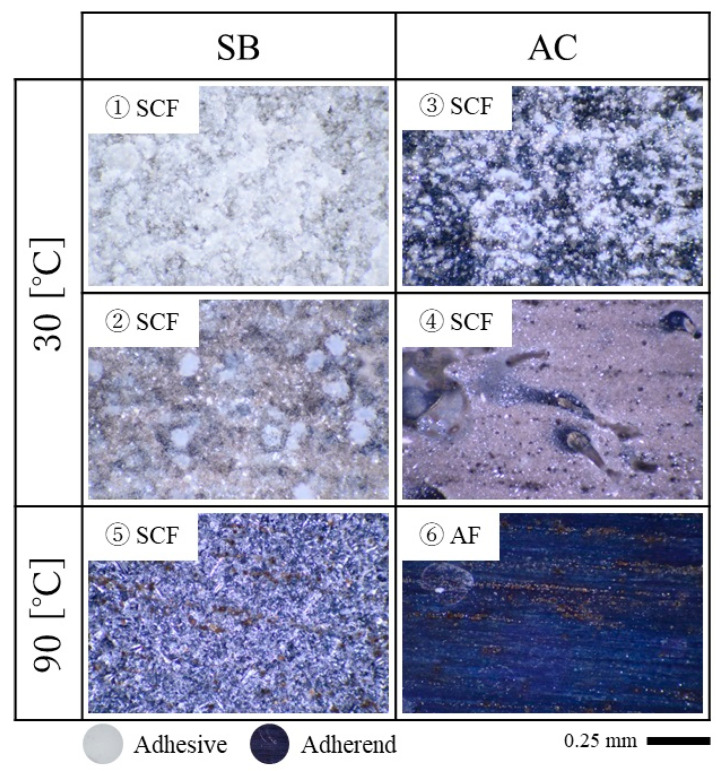
Magnified photograph under a microscope at selected points from Figure 11 (SCF: special cohesive failure: AF: adhesive failure).

After the tests, the specimens were completely separated, and fractured surfaces were observed, as shown in Figure 11. The initial crack positions are indicated by the white triangles, and the crack positions after the creep immersion tests are indicated by red triangles. Macroscopic observation of the surface showed that most of the epoxy remained on one side of the surface, and it appears as if the adhesive failure (AF) occurred in all tests. Here, AF is a failure mode of an adhesive layer when a failure occurs at the interface between the adhesive and either of the two adherends. Therefore, as shown in Figure 12, magnified observations were made at six specific points using a polarizing microscope (BX53P, Olympus Corp., Tokyo, Japan). Because the epoxy resin is white in color and aluminum alloy is dark in color when viewed with the polarizing microscope, it can be seen that some of the epoxy resin remained on the surfaces and that there were differences in the amount of epoxy resin remaining. In most cases, a thin layer of epoxy was seen on the surface, and the failure mode was considered substrate-near cohesive failure rather than AF. Thus, it is commonly classified as special cohesive failure (SCF). Here, cohesive failure (CF) is a failure mode of an adhesive layer when a failure occurs inside the layer and is classified as SCF, especially when a large amount of adhesive remains on either surface of the adherends. A detailed surface analysis showed that the sandblasting left more epoxy on the surface than the pickling, and AF was observed only when the pickling-treated specimen was immersed at 90 °C. In general, AF exhibits weak interfacial bonding. However, the crack propagated more in the sandblasted specimen with SCF than in the pickled specimen with AF at 90 °C immersion. In hot water, epoxy undergoes various chemical changes, and cracks can be caused by factors other than water penetration. Such complications may have reversed the trend.

It is also noteworthy that two steps of crack propagation were observed in both surface treatments with 32 °C immersion (SB1 and AC1). When comparing points 1 and 2 or 3 and 4, as shown in Figure 12, differences in the amount of epoxy remaining were observed depending on the location. The light-green triangles in Figure 11 show the transition point of failure, which was approximately 70 mm for SB1 and 65 mm for AC1. In the first step, a lot of epoxy remained on the aluminum surfaces, but with longer immersion time, the amount of epoxy remaining on the surface decreased. Along with this change, the crack propagated further, as shown by the arrows in Figure 9.

### 4.3. Creep Crack Growth Rate

The relationship between the crack growth rate and fracture parameters is used to discuss the crack growth behavior of the adhesive or interface under creep-loading conditions [8,42,43,44,45,46,47,48]. Similar to Paris’ law of fatigue crack growth (FCG) behavior, a power law relationship:

(7) $$\frac{da}{dt}=A{\left(\frac{G_{I}}{G_{\mathrm{IC}}}\right)}^{m}$$

is obtained for the CCG behavior when the adhesive is assumed to be a viscoelastic material, where A and m are constants, and t is time [42]. In fatigue testing, data smoothing of the FCG rate is required because of data scattering, and the incremental polynomial method and power-law fitting approach are commonly used [49]. The same is true for the CCG rate. Therefore, in this study, a linear approximation was made for every five consecutive data points of crack length versus time, and the slope was used as the differential coefficient of the central point.

**Figure 13:**
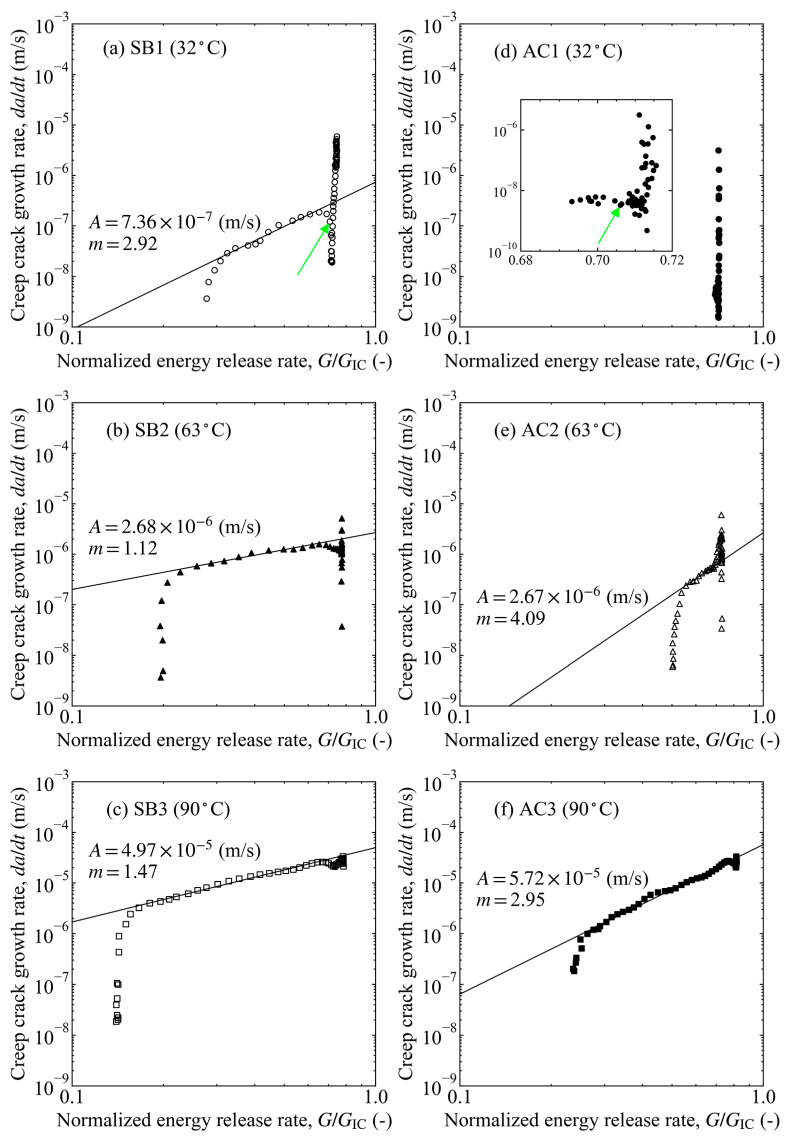
Creep crack growth relationship between the creep crack growth rate and normalized energy release rate at different temperatures and surface treatments: (**a**–**c**) sandblasting; (**d**–**f**) pickling at 32, 63, and 90 °C, respectively. A magnified graph is included in (**d**). The light-green arrows in (**a**) and (**d**) are the failure mode changing points, which relate to the locations of the light-green triangles in Figure 11.

**Figure 14:**
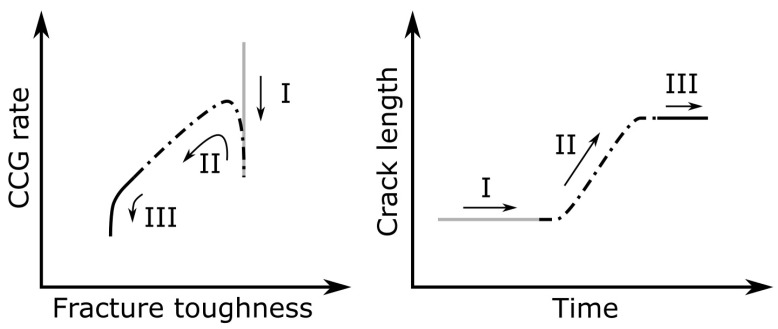
Schematic diagram of the state changes accompanying crack growth due to the creep immersion.

The CCG relationship (i.e., the CCG rate versus normalized energy release rate) is shown in Figure 13. Three characteristic trends were observed: initial stagnation, crack propagation, and threshold onset, as shown in Figure 14. In the case of immersion at 32 and 60 °C, the crack hardly grew for a while after loading, and the energy release rate was almost constant. Therefore, a vertical change was observed in the initial stage of the CCG relationship (region I). In contrast, for the 90 °C immersion, hardly any change was observed in the vertical direction, and the crack started to propagate immediately after loading, i.e., region II started without region I. Even at lower immersion temperatures, the crack started to propagate after a sufficient time had passed. In this case, the results follow the power law relationship after the CCG rate recovery, i.e., it flips at the turning point from regions I to II and after a while moves to the bottom left. The delay in the crack growth is considered, because it takes longer for water to penetrate the adhesive at lower temperatures. The penetration rate was faster at a hotter immersion, resulting in a difference in the initial trend. The arrows in Figure 13a,d refer to the light-green triangles in Figure 11, so the change in the fracture surfaces was related to the change in the CCG relationship. Pickling has a larger exponent in the power law m than sandblasting, and it is larger with a lower immersion temperature. Thus, it can be seen that the crack grew more slowly with a larger m. Comparing coefficient A, it increased with increasing temperature, but dependence on the surface treatment method was not observed. After region II, a vertical change associated with the slowing of the crack growth was observed (region III). Therefore, with the help of the CCG relationship, it is possible to clearly determine whether the results are approaching the threshold.

## 5. Conclusions

In this study, a DCB specimen consisting of aluminum plates bonded with an epoxy adhesive was set in a spring-loaded jig and subjected to a creep load. Degradation due to the creep and immersion was experimentally investigated by placing the loaded specimens in temperature-controlled water. In the original DCB test method, three parameters are needed to calculate the energy release rate: the crack length, load, and displacement. However, by introducing a data-reduction scheme using the compliance method, the energy release rate can be calculated with two parameters. Moreover, the relationship between displacement and load can be theoretically derived for the spring-loaded DCB test method when inserting a spring with a known spring constant. Therefore, another data-reduction scheme is possible, and it has been experimentally confirmed that only a single parameter, the load or displacement, is sufficient to calculate the energy release rate of the spring-loaded DCB tests. By changing the spring constant and the initial value of the load at a constant immersion temperature, it became clear that the threshold of the energy release rate depended only on the temperature. Conversely, the threshold value increased at lower immersion temperatures. Moreover, the crack growth was inhibited more by the pickling treatment than by the sandblasting treatment under a combination of creep and immersion conditions. The change in the crack growth behavior was evident when the results were plotted using the relationship between the creep crack growth rate and energy release rate. From various points of view, the accuracy of the spring-loaded DCB test was shown to be very high, and it became clear that it is a well-suited test method for evaluating hydrothermal creep.

## Figures and Tables

**Table 1:** Experimental conditions for studying the effects of the spring constant and initial energy release rate.

No.	$k_{\mathrm{spring}}$ (N/mm)	$G_{\mathrm{ini}}$ (J/m^2^)	Surface Treatment	T (°C)
1	25	500	Sandblasting	90
2	49	500		
3	98	498		
4	49	621		
5	49	372		
6	49	253		

**Table 2:** Experimental conditions for studying the effects of the surface treatment and immersion temperature.

Specimen	$k_{\mathrm{spring}}$ (N/mm)	$G_{\mathrm{ini}}$ (J/m^2^)	Surface Treatment	*T* (°C)
SB1	25	475	Sandblasting	32
SB2		504		63
SB3		500		90
AC1	25	465	Pickling	32
AC2		492		63
AC3		539		90

## Data Availability

The data presented in this study are available upon request from the corresponding author.
